# 2020年毛细管电泳技术年度回顾

**DOI:** 10.3724/SP.J.1123.2021.03024

**Published:** 2021-06-08

**Authors:** Bo WEI, Yao MA, Wenzhe TIAN, Xinying ZHAO, Feng QU

**Affiliations:** 1.北京理工大学生命学院, 北京 100081; 1. School of Life Science, Beijing Institute of Technology, Beijing 100081, China; 2.北京电子科技职业学院, 北京 100176; 2. Beijing Polytechnic College, Beijing 100176, China

**Keywords:** 毛细管电泳, 2020, 年度回顾, capillary electrophoresis (CE), 2020, annual review

## Abstract

该文为2020年毛细管电泳(capillary electrophoresis, CE)技术年度回顾。归纳总结了以“capillary electrophoresis-mass spectrometry”或“capillary isoelectric focusing”或“micellar electrokinetic chromatography”或“capillary electrophoresis”为关键词在ISI Web of Science数据库中进行主题检索得到的2020年CE技术相关研究论文222篇,以及中文期刊《分析化学》和《色谱》中CE技术相关的研究论文37篇。对2020年影响因子(IF)≥5.0的*Analytical Chemistry*, *Food Chemistry*, *Analytica Chimica Acta*和*Talanta*等13本期刊的38篇文章报道的科研工作作了逐一介绍;对IF<5.0的期刊中CE技术报道较为集中的*Journal of Chromatography A*和*Electrophoresis*两本分析化学类期刊发表40篇文章中的代表性内容作了综合介绍;对重要的中文期刊《分析化学》出版的“核酸适配体专刊”和《色谱》出版的2期CE技术专刊所收录的37篇文章中的工作作了总体介绍。总体来说,2020年CE技术发展趋势仍以毛细管电泳-质谱(CE-MS)的新方法和新应用最为突出,主要集中在CE-MS与电化学检测、固相萃取以及多种毛细管电泳模式的联用方面,CE-MS接口相关的报道较前几年有所减少;常规CE技术则以胶束电动毛细管色谱(MEKC)在复杂样本分析、浓缩富集应用为主,尤其在食品和药品等复杂基质样本分析方面的报道较为集中;此外,我国CE相关领域专家学者的科研成果涵盖了CE在生命科学、临床医学、医药研发、环境科学、天然产物、食品分析等领域的应用,代表了国内CE科研应用水平和现状。

## Abstract (en)

This paper reviews the developments in capillary electrophoresis (CE) in 2020. A total of 222 research papers related to CE technology published in 2020 were retrieved from the ISI Web of Science. These papers were selected by using the keywords “capillary electrophoresis-mass spectrometry”, “capillary isoelectric focusing”, “micellar electrokinetic chromatography”, or “capillary electrophoresis” as search conditions. A further 37 research papers related to CE technology were found in Chinese journals: *Chinese Journal of Analytical Chemistry* and *Chinese Journal of Chromatography*. The 38 papers published in 13 different journals with an impact factor (IF)≥5.0 are individually discussed. In particular, the journal Analytical Chemistry (IF=6.8) was found to have nine relevant articles, eight of which were related to CE-MS technology and its application, while *Food Chemistry* (IF=6.3) published five articles with four papers based on micellar electrokinetic chromatography (MEKC) and its combined technology. This demonstrated that MEKC can simultaneously separate negatively charged, positively charged, and neutral particles, in addition to featuring the advantage of combining different detector applications for the analysis of target matter in a complex matrix. In *Analytica Chimica Acta* (IF=6.0), five articles were collected, which mainly focused on new applications of CE-MS and multimode CE research. In *Talanta* (IF=5.3), 10 articles were collected, which focused on new methods and applications of CE and CE-MS. The remaining nine articles, i. e., seven experimental articles, one review article, and one introduction of scientific research achievements, were published in various other high-impact journals. At the same time, 184 articles were found in 89 journals with IF<5.0. In particular, the *Journal of Chromatography A* (IF=4.1) and *Electrophoresis* (IF=3.1) contained intensive reports for CE: 16 and 24 publications in relevant areas. The representative contents of 40 articles published in two journals were introduced comprehensively in this review, including new materials and new applications of MEKC, as well as new applications of CE-MS and CE. An overview of the work in the 37 articles collected from Chinese journals included “Aptamer Special Issue” published by *Chinese Journal of Analytical Chemistry* and two CE technical special issues published by *Chinese Journal of Chromatography*. In the “Aptamer Special Issue”, Qu Feng’s research group from Beijing Institute of Technology reported five works on aptamers based on CE, while the contents of *Chinese Journal of Chromatography* include new applications of CE and new materials in capillary tubes. In summary, new methods and applications of capillary electrophoresis-mass spectrometry (CE-MS) were the prominent development trend in CE technology in the year 2020, with the main focus being on the combination of CE-MS with electrochemical detection, solid-phase extraction, and various capillary electrophoresis modes. The number of reports related to the CE-MS interface has decreased compared to that in previous years. Conventional CE technology is mainly focused on the applications of MEKC in the analysis and concentration of complex samples, especially for the analysis of complex matrix samples such as food and drugs. In addition, the scientific research achievements of Chinese experts and scholars in CE-related fields cover the application of CE in life sciences, clinical medicine, pharmaceutical research and development, environmental science, natural products, food analysis, etc., representing the level and current status of the application of CE research in China.

截至2020年12月31日,以“capillary electrophoresis-mass spectrometry”或“capillary isoelectric focusing”或“micellar electrokinetic chromatography”或“capillary electrophoresis”为关键词在ISI Web of Science数据库中进行主题检索,检索到期刊论文共计222篇。

如图1所示,按照2020年影响因子(IF)划分,IF≥5.0的期刊共有13本,发表文章38篇,其中分析化学学科影响力较大的期刊*Analytical Chemistry*(IF=6.8)发表9篇,*Food Chemistry*(IF=6.3)发表5篇,*Analytica Chimica Acta*(IF=6.0)发表5篇,*Talanta*(IF=5.3)发表10篇,其他期刊9篇。IF<5.0的期刊共有89本,发表文章184篇,其中作为CE主要阵地的期刊*Journal of Chromatography A*(IF=4.1)和*Electrophoresis*(IF=3.1)分别发表16篇和24篇。此外,2020年《分析化学》出版了“核酸适配体专刊”,其中基于CE在核酸适配体筛选方面的报道有5篇,《色谱》出版了CE技术专刊2期(“毛细管电泳专辑(上、下)”),收录毛细管电泳相关文章32篇。

**图 1:**
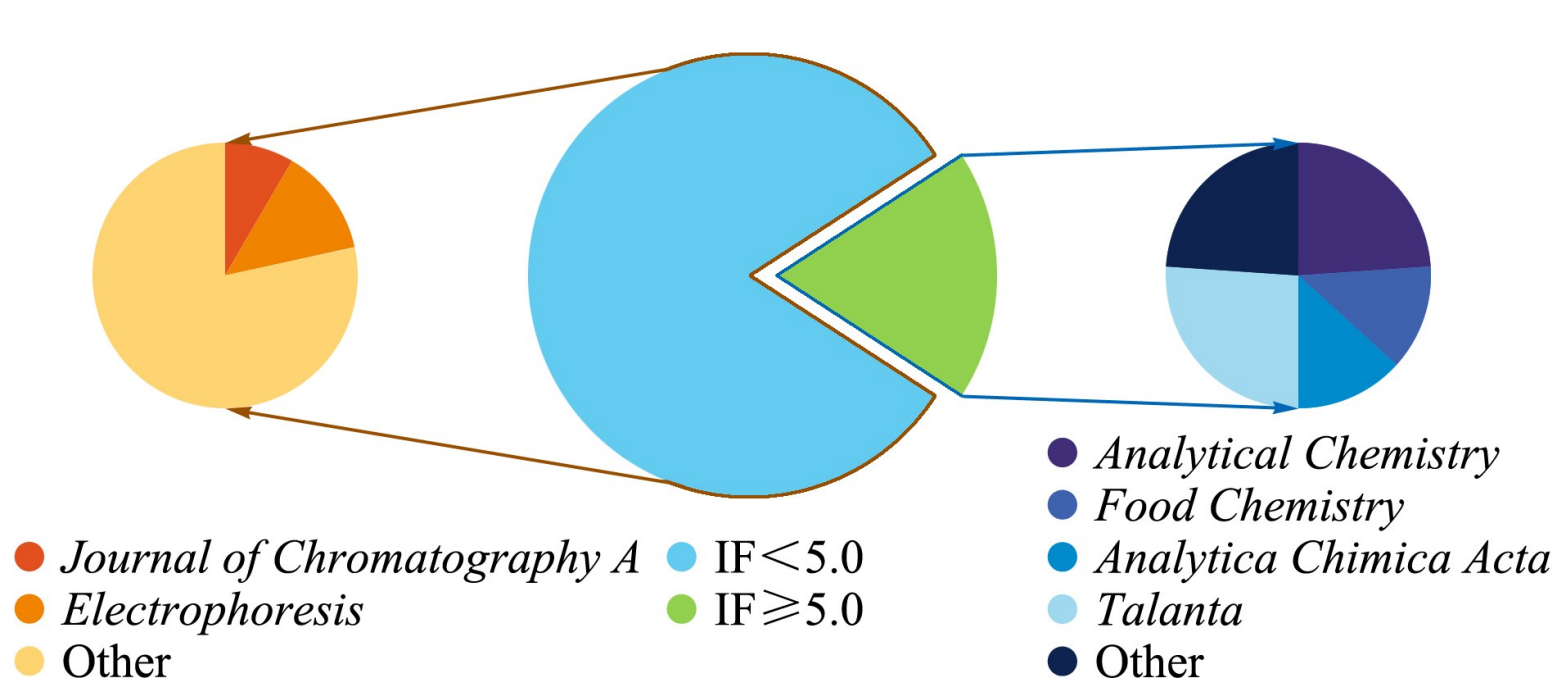
2020年CE文章发表情况

本文根据期刊发表论文情况,针对CE技术的代表性工作进行说明。

## 1 IF≥5.0期刊

### 1.1 *Analytical Chemistry*

发表的9篇论文,8篇均涉及CE-MS技术及其应用。Drouin等^[1]^利用北美洲和欧洲13个实验室中的16个CE-MS平台提供的有效电泳迁移率(μeff)作为关键参数,评估CE-MS的重现性和鉴定能力的工作,证明了CE-MS是一种可行的代谢组学方法。Courtney等^[2]^提出了一种新的自由流逆流梯度聚焦(FF-CGF)机制,通过从自由流电泳(FFE)腔室的侧壁引入流体流而产生逆流梯度,以平衡电泳迁移;与自由流区带电泳(FFZE)相比,分辨率将较少受样品注入宽度和分离区域大小的影响,因此可以减少设备占用空间,实现简单集成和降低使用功耗;与自由流等电聚焦(FF-IEF)相比,在原理上相似,但聚焦点由分析物的电泳迁移率决定;随着FFE与质谱学联用的应用越来越多,FF-CGF的宽松缓冲范围将更符合人们的实际需求。

1.1.1 CE-MS与其他技术的联用

Herl等^[3]^利用电化学-毛细管电泳-质谱(EC-CE-MS)法研究胸腺嘧啶在丝网印刷碳电极上的电氧化,通过电化学预处理样品在线表征的方法,可以获得电化学质谱数据,还可以进一步理解电氧化过程。Gascon等^[4]^提出在线适配体亲和固相萃取毛细管电泳质谱法(AA-SPE-CE-MS),一步实现血液中*α*-突触核蛋白(*α*-syn)的纯化、预浓缩、分离和表征,LOD为0.2 μg/mL,是CE-MS(LOD=20 μg/mL)的1/100。Stolz等^[5]^使用5层连续的多种离子聚合物涂层覆盖的毛细管来实现高效分离,可完整地分离血红蛋白(Hb)糖基化的α链和β链的位置异构体;涂覆的DEAEDq-PMA(DEAEDq: quarternized diethylaminoethyl dextran; PMA: poly-(methacrylic acid))涂层具有蛋白质吸附少、涂层稳定性高、MS兼容性好、电渗流(EOF)温和的特性。Delvaux等^[6]^研究了具有相同序列并带有两个分子内二硫键但二硫键连接方式不同的肽在毛细管区带电泳质谱(CZE-MS)和离子淌度质谱(IM-MS)中的分离效率,结果表明CZE-MS适用于有限数量的样品分析;而使用具有更高解析度的最新一代离子迁移质谱仪时,IM-MS方法可用于高通量筛选二硫键。

1.1.2 多种毛细管电泳模式与MS联用

Salplachta等^[7]^结合瞬时等速电泳(tITP)和胶束电动色谱(MEKC),通过基质辅助激光解吸/电离飞行时间质谱(MALDI-TOF MS)鉴定分生孢子;首先,对毛细管进行修饰,即将分生孢子动态附着在通过超临界水(SCW)蚀刻制备的熔融石英毛细管内表面的粗糙部分上,然后使用tITP和MEKC的组合将附着的分生孢子解吸、浓缩和分离并通过MALDI-TOF MS进行分析,LOD为20分生孢子/mL,有助于快速诊断病人是否感染并及时治疗。Xu等^[8]^使用毛细管等电聚焦-串联质谱(cIEF-MS/MS),从大肠埃希氏菌蛋白质组中鉴定出711种蛋白质,而将尺寸排阻色谱(SEC)-cIEF与ESI-MS/MS偶联可从大肠杆菌蛋白质组中鉴定出近2000种蛋白质,结果表明cIEF-MS/MS是自上而下的蛋白质组学(TDP)的有力工具,能描述复杂蛋白质组中蛋白质形式。

1.1.3 接口改进

Kristoff等^[9]^建立了一种基于振动尖锐边缘喷雾电离(VSSI)新接口的CE-VSSI-MS方法,其中CE-VSSI接口可以保持CE的高分离效率,且与不同的挥发性背景电解质和离子强度兼容,并能够在接近中性的pH下分离,不需要有机改性剂来辅助液滴形成。

### 1.2 *Food Chemistry*

发表5篇论文,其中4篇基于MEKC及其联用技术,充分体现了MEKC可以同时分离带负电、正电和电中性粒子的特点,以及结合不同检测器应用对复杂基质中目标物分析的优势。Rezende等^[10]^通过微芯片胶束电动色谱法检测威士忌酒样品中的酒精含量,通过电容耦合非接触式传导检测(C^4^D)法检测MEKC模式电泳芯片上醇的分离,由于其分析时间短、线性良好(*R*^2^=0.98)、LOD低(0.5%, v/v),因此在酒精饮料质量控制中有极大的应用潜力。Sun等^[11]^用基于电压程序(VP)和激光诱导荧光(LIF)检测器的MEKC法定量分析实际样品中的天然辣椒素(CAP)和二氢辣椒素(DHC),在不降低解析度的前提下分离时间显著缩短,同时,LIF可以提升检测灵敏度,从而快速、灵敏地检测各种食品中的CAP和DHC;该法成功应用于辣椒、白菜和泡菜中CAP的定量测定,置信度为98%,具有成为检测商业辣椒样品、食品、废油、细胞、制药和调味品行业中CAP和DHC工具的潜力。Gonzalez等^[12]^利用MEKC-MS测定辣椒制品中苏丹I、苏丹II、苏丹III和苏丹IV染料;用乙腈从辣椒样品中提取目标化合物并通过冷冻纯化提高提取效率,减小基质效应;方法线性良好(*R*^2^≥0.9914), LOD低(22 μg/kg);使用这种方法对20种辣椒产品进行分析,证明该方法在食品质量和安全控制方面具有适用性。Yu等^[13]^基于深共熔溶剂与MEKC结合的盐析辅助分散液-液微萃取法(SO-DLLME-DES-BE-MECC),测定水、蜂蜜和牛奶中的氟喹诺酮类药物,线性良好,且LOD小于0.010 μg/mL;水、蜂蜜和牛奶样品的回收率分别为95.0%~104.9%、90.1%~110.2%和87.8%~114.1%,是食品和水样中痕量分析的可用替代方法。

此外,Ogura等^[14]^基于代谢组学,利用CE-MS和LC-MS分析了用玉米或者大米喂养的两类母鸡发生鸡蛋和煮鸡蛋的冷冻干燥样品,探究加热和饲料作物对游离氨基酸和糖等味道成分的影响;发现与玉米喂养相比,大米喂养母鸡的蛋黄和蛋白中甜菜碱和尿苷一磷酸浓度分别高出3倍,煮熟的蛋白所含氨基酸和果糖的浓度比未煮过的蛋白高4倍以上,煮熟后蛋黄中的代谢物变化很小。

### 1.3 *Analytica Chimica Acta*

发表5篇CE相关的方法及应用研究论文,主要工作为CE-MS的新应用和多模式CE研究。Krenkova等^[15]^设计并合成了基于多阳离子氨基芘的寡糖标记标签,标记的麦芽低聚糖进行CE-MS分析时有亚微摩尔检测限,表明新标签可作为寡糖分析的有力工具。GstCottner等^[16]^通过无鞘流CE-MS法对双特异性抗体(BsAbs)进行完整抗体和亚基特异性分析,无鞘流接口连接可以提高电离效率和灵敏度,表征自由的轻链、同型和异型二聚体,显示了无鞘流CE-MS表征BsAbs的潜力。Gascon等^[17]^提出带有纳升阀(nv)的固相萃取-毛细管电泳-质谱(nvSPE-CE-MS),可以避免基质吸附,并实现背景电解质兼容,能检测标准品和血浆样品中的阿片类和淀粉样β肽生物标志物;LOD是CE-MS的约1/200,表明nvSPE-CE-MS能分析复杂样品中低浓度分析物。Peng等^[18]^开发了一种在线环糊精(CD)辅助胶束推扫电动色谱法(sweeping-MEKC),分离和浓缩铁皮石斛中的4种中性分析物,线性良好,LOD为13~40 ng/mL,该法的效率和浓度敏感性高,适用于分离和浓缩实际样品中的中性分析物。Chau等^[19]^将电容耦合非接触电导率检测(C^4^D)与微流控毛细管等电聚焦(mCIEF)结合,测定了3种荧光蛋白质的电导率,平均分离分辨率为2.06;该装置可以扩大可用的pH范围,并用于分析具有诊断意义的非荧光蛋白质,还可以进行便携分析。

### 1.4 *Talanta*

发表10篇论文,其中6篇涉及CE-MS, 4篇涉及CE,研究内容集中在CE和CE-MS的新方法和新应用。

CE-MS Vermeire等^[20]^构建了一种新型的无鞘流毛细管电泳-电喷雾电离-质谱(CE-ESI-MS)接口,用于小分子和生物分子分析,与已报道的接口相比,新型无鞘流CE-ESI-MS接口具有多个优点:接口牢固、不需要额外的干燥气体、传输效率高等。Domínguez-Álvarez^[21]^采用分散液-液微萃取法处理样品,通过CE-ESI(-)-MS检测饮用水中的无机砷,可以快速测定总无机砷含量。Segers等^[22]^借助CE-MS,使用6 Hz电刺激引发癫痫的小鼠模型分析在血浆中诱发性癫痫的代谢产物,证明了使用CE-MS可以分析体液内与癫痫发作活动有关的代谢产物。Arteu等^[23]^提出二氧化钛基质固相萃取-在线毛细管电泳-质谱法(TiO_2_-SPE-CE-MS),分析胰蛋白酶消化物中获得的糖肽糖型,检测限是CE-MS的1/100,可应用于蛋白质消化物分析或糖蛋白组学的比较研究。Petrov等^[24]^使用CZE和电喷雾质谱法分析了市场购买的460种代谢物,确定了276种代谢物的迁移率值。Vásconez等^[25]^对比了两性离子-亲水相互作用毛细管液相色谱(capZIC-HILIC)和CE,发现capZIC-HILIC-UV和CE-UV在线性范围内的LOD和分离分辨率相似,但通过CE-UV获得了更高的分离效率;当与MS检测器连接时,capZIC-HILIC-MS的LOD低于CE-MS(约1/5), CE和capZIC-HILIC两种方法对样品检测都具有巨大潜力。

CE Tuma等^[26]^用聚(丙烯酰胺-3-丙烯酰胺基丙基三甲基氯化铵)共聚物(PAMAPTAC)共价涂覆熔融二氧化硅毛细管,得到一种可调逆电渗流的涂层毛细管;当PAM涂层中APTAC离子含量从0%提高到6%时,氯胺酮和去甲氯胺酮的分离度可从0.8提高到3,并且APTAC离子含量的变化可在较广的迁移率范围内产生阴离子EOF,在逆电渗流电泳方案中,可明显提高相邻峰的分离度,降低了对临床样品的实验室处理要求。Yu等^[27]^使用CTAB和熔融石英毛细管在开管小管电色谱(OT-AMEC)中分离阳离子和阴离子分析物,并估算了分析物保留因子(*k*)值的方程式。Chen等^[28]^利用不连续整体式固定pH梯度毛细管等电聚焦(DM-IPG-CIEF)富集pI在6.0~9.0范围内的外泌体蛋白质,通过nano-LC-MS/MS鉴定了该范围内的131种外泌体蛋白质,结果表明DM-IPG-CIEF系统具有高的预分离能力。Pieckowski等^[29]^将大容量样品堆积与极性切换和环糊精电动色谱法(LVSS-PS-CDEKC)组合,用于预浓缩和分离测定8种防腐剂,LOD在0.8~5 ng/mL范围内,是未使用大容量样品堆积技术的500倍左右。

### 1.5 其他IF≥5.0期刊

其他IF≥5.0的期刊共发表CE相关文章9篇,其中*Small*影响因子最高(IF=11.5);研究内容主要集中在CE技术在医学领域的应用,包括实验类文章7篇、综述类文章1篇、科研成果介绍1篇。

IF≥9.0 综合类期刊*Small*发表Chetwynd等^[30]^基于CE-MS的代谢组学方法测定纳米材料(NMs)代谢物电晕(纳米材料在生物系统中迅速被生物分子包覆,形成电晕)的研究,使用不含蛋白质的血浆样品和完整血浆样品定量比较了代谢产物电晕的组成,结果表明样品中的蛋白质对代谢产物电晕影响很大;该工作为将纳米材料的代谢产物电晕纳入未来的纳米安全性考察奠定了基础。医学期刊*PLoS Medicine*(IF=10.5)发表Mahajan等^[31]^在脑组织样品中应用CE-MS与基因表达分析相结合的靶向代谢组学方法,测定脑组织中26种代谢产物的浓度,研究阿尔茨海默病(AD)的发病机理;结果表明细胞甲基化电位的改变和转甲基化途径中通量的增加、多胺生物合成和分解增加以及神经递质代谢异常与AD病理学的严重程度及临床症状的表达有关;研究结果增强了对AD发病机理代谢基础的进一步了解。综述型期刊*TrAC*-*Trends in Analytical Chemistry*(IF=9.8)发表Bermejo等^[32]^的综述,概述了手性毛细管电泳的基本原理、特点、研究进展及发展趋势。

9.0≥IF≥5.0 *Mass Spectrometry Reviews*(IF=8.9)发表Karger^[33]^介绍Paul Vouros在东北大学和Barnett研究所的多年科研工作成果。*Neuropathology and Applied Neurobiology*(IF=7.5)报道Zampar等^[34]^基于毛细管等电聚焦法研究血管淀粉样蛋白b(Ab)沉积物的N末端异质性,从而理解各种Ab变体在疾病发生和发展过程中的作用,以制定合适的靶标疗法。*Carbohydrate Polymers*(IF=7.2)报道Yu等^[35]^使用滤膜捕集富集法制备对抗凝血酶III(AT)具有亲和力的糖胺聚糖(GAG),并使用CZE- MS/MS测定结构。*Microchimica Acta*(IF=6.2)报道Horka等^[36]^结合CE和MALDI-TOF MS,监测体内的治疗性噬菌体,CE可以将噬菌体预浓缩至足够数量以便通过MALDI-TOF MS进行后续鉴定。*Soil Biology* & *Biochemistry*(IF=5.8)发表Warren^[37]^通过LC-MS鉴定并定量分析土壤样品中的极性脂质,并通过CE-MS定量释放的头基来获得独立估计值,结果表明贫磷土壤中不是减慢磷脂的合成和生长,而是表达无磷脂质(例如甜菜碱脂质)。*EBioMedicine*(IF=5.7)报道Bannaga等^[38]^通过CE-MS分析尿中低分子量蛋白质组,探讨尿肽对肝纤维化患者的诊断价值。

## 2 IF<5.0的重要期刊

### 2.1 *Journal of Chromatography A*

发表16篇论文,其中4篇涉及CE-MS,体现新材料的应用;另有12篇涉及CE,主要是MEKC模式的新应用。

CE-MS Zhang等^[39]^通过用氢氟酸将二氧化硅毛细管的出口端蚀刻到对称尖端并覆盖金箔,形成ESI电极作为无鞘接口,实现毛细管电泳与质谱的耦合;与气体沉积产生的金属涂层相比,金箔的机械强度和化学稳定性更高,更易于制备。Gou等^[40]^在CE-MS中通过动态pH结(DPJ)进行样品预富集,采用纳流鞘液接口(EMASS-II)提高样品装载量和灵敏度,并分析1 μg大肠杆菌蛋白质组消化物,获得了符合预期的序列覆盖率。Akter等^[41]^使用共价键合的2-丙烯酰胺基-2-甲基-1-丙烷磺酸(AMPS)色谱柱,通过MEKC-MS对3种β阻滞剂对映体进行分离检测,LOD为0.2 μg/mL,可以提升阴离子手性化合物分离的重复性。Pont等^[42]^以金纳米颗粒的聚合物整体材料(AuNP@monolith)作为微墨盒,通过固相萃取-在线毛细管电泳-质谱(SPE-CE-MS)分析人甲状腺素蛋白(TTR),并评估新型微墨盒的性能,其LOD是单纯的CE-MS的1/50(5 mg/L vs 250 mg/L)。

MEKC Li等^[43]^用聚吡咯包覆的磁性纳米粒子固相萃取4种*β*-内酰胺(奥沙西林(OXA)、克洛西林(CLOX)、双氯西林(DIC)和氟氯西林(FLU)),通过胶束电动毛细管色谱-二极管阵列检测(MEKC-DAD), OXA、CLOX、FLU的LOD为1.0 μg/L, DIC的LOD为0.8 μg/L,该方法可用于水样中*β*-内酰胺的测定。Salido-Fortuna等^[44]^第一次报道了基于羟丙基-*β*-环糊精与四丁基铵-L-赖氨酸和四丁基铵-L-谷氨酸结合使用的两种手性离子液体通过MEKC测定益康唑和舒康唑,LOD低于1.6 mg/L, LOQ低于5.3 mg/L,能够确定药物制剂中舒康那唑对映体。Zhen等^[45]^将电动注射辅助胶束环糊精堆积(MCDS)与MEKC结合,在线浓缩三嗪类除草剂,线性范围为0.1~20 μg/mL(*R*^2^≥0.9985),是三嗪类除草剂痕量分析的有效方法。Liu等^[46]^将场增强的样品注射与MCDS-MEKC结合,测定大枣糖苷A和B, LOD为0.2~0.3 μg/mL(*R*^2^>0.999),可用于分析复杂样品基质中的中性分子。

### 2.2 *Electrophoresis*

发表24篇论文,其中CE-MS方法研究及应用12篇,多模式CE的应用12篇。

CE-MS Zhang等^[47]^通过无鞘CE-MS在阳离子模式下使用ESI分析哺乳动物细胞中的核苷酸,进样量为6.5 nL时,检出限范围为0.06至1.3 nmol,该方法可用于少量生物材料的代谢谱分析。Hocker等^[48]^开发了一种新型纳流鞘液CE-MS接口,更方便安装,并且发射器尖端寿命更长,同时保持了纳流CE-MS联轴器的灵敏度。Hlozek等^[49]^利用CE-MS方法监测人血清中的利奥西呱和去甲基利奥西呱水平,在10~1000 ng/mL范围内线性良好,准确度90.1%~114.9%,证明CE-MS是一种灵敏的分析工具。Yan等^[50]^通过CZE-ESI-MS分析二甲基标记的复杂蛋白质组中的氘同位素效应,轻质标记肽相对重质标记肽的迁移时间偏移0.1 s,是峰宽的2.5%,而在HPLC-ESI-MS中迁移时间偏移3 s,大约是峰宽的一半。Wang等^[51]^开发了一种动态pH平衡连接聚焦的CE-MS/MS方法,用于定性分析氨基酸、肽和胰蛋白酶肽;在线聚焦提高了MS信号强度,扩大了分析的浓度范围,能获得更多的序列覆盖率数据。

CE Ren等^[52]^使用极性反转毛细管等电聚焦分析重组人促红细胞生成素(rhuEPO)的亚型;与CZE相比,这种方法具有更好的重复性和分辨率,还能提供每个亚型的pI值,有潜力成为rhuEPO异构体分析的替代方法。Li等^[53]^使用MEKC-UV测定化妆品中的4种萘二醇,LOD为0.070~0.19 μg/mL, LOQ为0.23~0.63 μg/mL;与HPLC-UV方法相比,MEKC法检测时间更短,成本更低。Shieh等^[54]^通过环糊精-胶束电动色谱(CD-MEKC)法研究了人极低密度脂蛋白(VLDL)的载脂蛋白(APO),在0.01~0.54 mg/mL范围内线性良好,LOD低于0.02 mg/mL;比较了尿毒症患者和健康受试者的VLDL、APO, CD-MEKC谱有显著差异,利用该方法有助于了解未来尿毒症和心血管疾病(CVD)的发展。Zhang等^[55]^报道了一种结合甘氨酸和牛磺酸的新型基质配方,可以显著改善聚乙二醇化蛋白中电荷变体的分离,从而可以对聚乙二醇化的蛋白质进行毛细管等电聚焦成像。

## 3 代表性的中文期刊

### 3.1 《分析化学》

《分析化学》出版的“核酸适配体专刊”中,北京理工大学屈锋课题组发表了5篇基于CE筛选核酸适配体的工作。朱超等^[56]^概述了CE在核酸适配体筛选中的应用、不同靶标的筛选策略和多种筛选模式。杨歌等^[57]^基于毛细管电泳-指数富集配体系统进化技术(CE-SELEX)筛选人免疫球蛋白(IgG)的Fc片段核酸适配体,在优化条件下,经3轮筛选直接获得Fc片段的特异性核酸适配体,此适配体有望用于IgG的效应功能调控。李林森等^[58]^研究了CE筛选核酸适配体过程中核酸库容量对筛选效率的影响,结果表明,大容量核酸库中筛选的核酸适配体序列与小容量核酸库筛选的亲和力相当,核酸库容量对适配体筛选效率没有显著影响。孙淼等^[59]^筛选了钙网蛋白(CRT)的核酸适配体Apt 23,对CRT的亲和力和特异性良好,可作为血清基质中CRT的检测探针和乳腺癌细胞的成像探针。杨歌等^[60]^以脱铁转铁蛋白(A-TF)为模式蛋白,利用CE方法研究核酸库长度、孵育温度、缓冲溶液的种类与pH值、金属离子等因素对靶蛋白与核酸库相互作用的影响。

### 3.2 《色谱》

《色谱》出版了“毛细管电泳专辑(上、下)”,共收录文章32篇。专刊采编了国内CE领域专家学者的用心之作,涵盖了CE在生命科学、临床医学、医药研发、环境科学、天然产物、食品分析等领域的应用,代表了国内CE科研应用水平和现状。

CE新应用 孟庆威等^[61]^基于CE-LIF和CE-前沿分析(FA)方法,研究适配体-靶分子间的亲和作用;结果表明,CE-FA法可相对有效地克服高压电场对复合物稳定性的影响,具有适用范围广、方法稳定、结果拟合简便准确等特点。王双双等^[62]^采用压力辅助毛细管电泳前沿分析(PACE-FA)结合ESI-MS研究人类癌基因c-myb启动子G-四链体(G4)与天然产物分子间的相互作用,该组合方法不仅分析速度快,而且能够提高亲和分析的准确度和特异性。王伟峰等^[63]^开发了一种MEKC法用于快速检测玫瑰纯露中的指标成分苯乙醇,为玫瑰纯露及其制品的质量控制提供了一种简便、快速、灵敏的分析方法。沈煜婷等^[64]^开发了一种CE检测肝素和低分子量肝素的平均硫酸化程度的方法,用于不同厂家生产的依诺肝素平均硫酸化程度比较,该方法在肝素类药物生产过程的质量控制中具有良好的应用潜力。

新材料在毛细管修饰中的应用 张淼等^[65]^制备了一种对溶菌酶具有可控吸附性能的混合刷涂层毛细管;该涂层的制备只需一步,而且具有很好的稳定性,实现毛细管电泳在线富集溶菌酶,提高检测灵敏度,为毛细管电泳分析痕量蛋白质提供了一种简单有效的方法。赵凌艺等^[66]^制备了一种二维吖嗪共价有机骨架材料(ACOF-1)作为固定相的ACOF-1涂层毛细管,证明了将其用于开管-毛细管电色谱(OT-CEC)分离检测硝基苯酚类环境内分泌干扰物(EEDs)的可行性。刘丽丽等^[67]^合成得到两亲性嵌段聚合物-聚(苯乙烯-甲基丙烯酸缩水甘油酯)(P(St-GMA)),并将其涂覆到毛细管内壁,通过OT-CEC分析3种解热镇痛药物,结果表明使用涂覆毛细管显著提升了解热镇痛药物的分离效率。

## 4 结语

2020年CE技术发展趋势仍以CE-MS的新方法和新应用最突出。常规CE技术则以MEKC在复杂样本分析、浓缩富集应用为主,尤其在食品和药品等复杂基质样本分析方面较为突出。《分析化学》和《色谱》等国内主要期刊集中发表了CE技术新方法和新应用专刊,呈现了近期我国科技工作者在CE方面的工作进展。

以上内容难免有遗漏和不妥之处,请广大学界同仁及应用从业人员批评指正。

## References

[1] Drouin N, Mever M V, Zhang W, et al. Anal Chem, 2020,92(20):14103 32961048 10.1021/acs.analchem.0c03129 PMC7581015

[2] Courtney M, Thompson E, Glawdel T, et al. Anal Chem, 2020,92(10):7317 32336087 10.1021/acs.analchem.0c01024

[3] Herl T, Matysik F M. I. Anal Chem, 2020,92(9):6374 32227929 10.1021/acs.analchem.9b05406

[4] Pero-Gascon R, Benavente F, Minic Z, et al. Anal Chem, 2019,92(1):1525 31825201 10.1021/acs.analchem.9b04802

[5] Stolz A, Hedeland Y, Salzer L, et al. Anal Chem, 2020,92(15):10531 32628011 10.1021/acs.analchem.0c01350

[6] Delvaux C, Massonnet P, Kune C, et al. Anal Chem, 2019,92(3):2425 10.1021/acs.analchem.9b03206 31885261

[7] Salplachta J, Horka M, Karasek P, et al. Anal Chem, 2020,92(11):7588 32384240 10.1021/acs.analchem.0c00165

[8] Xu T, Shen X, Yang Z, et al. Anal Chem, 2020,92(24):15890 33263984 10.1021/acs.analchem.0c03266 PMC8564864

[9] Kristoff C J, Li C, Li P, et al. Anal Chem, 2020,92(4):3006 31971372 10.1021/acs.analchem.9b03994 PMC7295075

[10] Rezende K C A, Martins N M, Talhavini M, et al. Food Chem, 2020,329:127175 32516708 10.1016/j.foodchem.2020.127175

[11] Sun Y, Park B, Ha J H, et al. Food Chem, 2020,323:126831 32334311 10.1016/j.foodchem.2020.126831

[12] Moreno-González D, Jáč P, Švec F, et al. Food Chem, 2020,310:125963 31838374 10.1016/j.foodchem.2019.125963

[13] Yu K, Yue M E, Xu J, et al. Food Chem, 2020,332:127371 32622188 10.1016/j.foodchem.2020.127371

[14] Ogura T, Wakayama M, Ashino Y, et al. Food Chem, 2020,327:127077 32485660 10.1016/j.foodchem.2020.127077

[15] Krenkova J, Liskova M, Cmelik R, et al. Anal Chim Acta, 2020,1095:226 31864627 10.1016/j.aca.2019.10.032

[16] Gstöttner C, Nicolardi S, Haberger M, et al. Anal Chim Acta, 2020,1134:18 33059862 10.1016/j.aca.2020.07.069

[17] Pero-Gascon R, Benavente F, Christian Neusü, et al. Anal Chim Acta, 2020,1140:1 33218471 10.1016/j.aca.2020.09.036

[18] Peng L Q, Dong X, Zhen X T, et al. Anal Chim Acta, 2020,1105:224 32138922 10.1016/j.aca.2020.01.037

[19] Chau M K, Arega N G, Tran N A N, et al. Anal Chim Acta, 2020,1124:60 32534676 10.1016/j.aca.2020.05.028

[20] Vermeire P J, Van Schepdael A, Petersen N J. Talanta, 2020,214:120853 32278416 10.1016/j.talanta.2020.120853

[21] Domínguez-Álvarez J. Talanta, 2020,212:120803 32113565 10.1016/j.talanta.2020.120803

[22] Segers K, Zhang W, Aourz N, et al. Talanta, 2020,217:121107 32498853 10.1016/j.talanta.2020.121107

[23] Mancera-Arteu M, Lleshi N, Sanz-Nebot V, et al. Talanta, 2020,209:120563 31892091 10.1016/j.talanta.2019.120563

[24] Petrov A P, Sherman L M, Camden J P, et al. Talanta, 2020,209:120545 31892063 10.1016/j.talanta.2019.120545 PMC6956853

[25] Vásconez J, Pero-Gascon R, Giménez E, et al. Talanta, 2020,219:121263 32887154 10.1016/j.talanta.2020.121263

[26] Tůma P, Koval D, Sommerová B, et al. Talanta, 2020,217:121094 32498904 10.1016/j.talanta.2020.121094

[27] Raymond B Y, Quirino J P. Talanta, 2020,208:120401 31816751 10.1016/j.talanta.2019.120401

[28] Chen H, Zhang L, Zhang W, et al. Talanta, 2020,208:119876 31816743 10.1016/j.talanta.2019.04.077

[29] Pieckowski M, Kowalski P, Bᶏczek T. Talanta, 2020,211:120673 32070558 10.1016/j.talanta.2019.120673

[30] Chetwynd A J, Zhang W, Thorn J A, et al. Small, 2020,16(21):2000295 10.1002/smll.202000295 32240572

[31] Mahajan U V, Varma V R, Griswold M E, et al. PLoS Med, 2020,17(1):e1003012 31978055 10.1371/journal.pmed.1003012 PMC6980402

[32] Bernardo-Bermejo S, Sánchez-López E, Castro-Puyana M, et al. TrAC-Trends Anal Chem, 2020,124:115807

[33] Karger B L. Mass Spectrom Rev, 2020,39(1/2):13 30845356 10.1002/mas.21593

[34] Zampar S, Klafki H W, Sritharen K, et al. Neuropath Appl Neuro, 2020,46(7):673 10.1111/nan.12637 PMC8082844 32497293

[35] Yu Y, Zhang F, Renois-Predelus G, et al. Carbohyd Polym, 2020,245:116623 10.1016/j.carbpol.2020.116623 PMC7387750 32718661

[36] Horká M, Karásek P, Šalplachta J, et al. Microchim Acta, 2020,187(3):1 10.1007/s00604-020-4154-6 32076849

[37] Warren C R. Soil Biol Biochem, 2020,140:107655

[38] Bannaga A S, Metzger J, Kyrou I, et al. EBioMedicine, 2020,62:103083 33160210 10.1016/j.ebiom.2020.103083 PMC7648178

[39] Zhang H, Lou C, Li J, et al. J Chromatogr A, 2020,1624:461215 32540065 10.1016/j.chroma.2020.461215

[40] Gou M J, Nys G, Cobraiville G, et al. J Chromatogr A, 2020,1618:460873 31987525 10.1016/j.chroma.2020.460873

[41] Akter F, Shamsi S A. J Chromatogr A, 2020,1617:460835 31928773 10.1016/j.chroma.2019.460835 PMC7160040

[42] Pont Villanueva L, Marin G, Vergara-Barberán M, et al. J Chromatogr A, 2020,1622:461097 32381302 10.1016/j.chroma.2020.461097

[43] Li X, Yin Z, Zhai Y, et al. J Chromatogr A, 2020,1610:460541 31564564 10.1016/j.chroma.2019.460541

[44] Salido-Fortuna S, Marina M L, Castro-Puyana M. J Chromatogr A, 2020,1621:461085 32376018 10.1016/j.chroma.2020.461085

[45] Zhen X T, Chen Y, Yang J, et al. J Chromatogr A, 2020,1628:461438 32822978 10.1016/j.chroma.2020.461438

[46] Liu C, Li J, Zhu L, et al. J Chromatogr A, 2020,1618:460854 31980258 10.1016/j.chroma.2020.460854

[47] Zhang W, Guled F, Hankemeier T, et al. Electrophoresis, 2020,41(5/6):360 31907937 10.1002/elps.201900417

[48] Höcker O, Knierman M, Meixner J, et al. Electrophoresis, 2021,42(4):369 32776368 10.1002/elps.202000169

[49] Hložek T, Štícha M, Bursová M, et al. Electrophoresis, 2020,41(18/19):1564 32640044 10.1002/elps.202000135

[50] Yan X, Sun L, Dovichi N J, et al. Electrophoresis, 2020,41(15):1374 32548848 10.1002/elps.202000051 PMC7540333

[51] Wang L, Cheng J, McNutt J E, et al. Electrophoresis, 2020,41(21/22):1832 32436592 10.1002/elps.202000076

[52] Ren T, Zhang X, Li X, et al. Electrophoresis, 2020,41(23):2055 32841408 10.1002/elps.202000165

[53] Li X, Zheng Z, Liu H, et al. Electrophoresis, 2020,41(23):1991 32839980 10.1002/elps.202000184

[54] Shieh Y T, Chang C T, Toh J J, et al. Electrophoresis, 2020,41(15):1333 32390137 10.1002/elps.202000065

[55] Zhang X, Chemmalil L, Ding J, et al. Electrophoresis, 2020,41(9):735 31967659 10.1002/elps.201900406

[56] Zhu C, Zhao X Y, Yang G, et al. Chinese Journal of Analytical Chemistry, 2020,48(5):573

[57] Yang G, Han S M, Zhao L P, et al. Chinese Journal of Analytical Chemistry, 2020,48(5):601

[58] Li L S, Zhu C, Zhao Y, et al. Chinese Journal of Analytical Chemistry, 2020,48(5):615

[59] Sun M, Yang G, Zhao Y, et al. Chinese Journal of Analytical Chemistry, 2020,48(5):642

[60] Yang G, Zhao Y, Han S M, et al. Chinese Journal of Analytical Chemistry, 2020,48(5):632

[61] Meng Q W, Guo L, Xie J W, Chinese Journal of Chromatography, 2020,38(9):1078 34213274 10.3724/SP.J.1123.2020.04001

[62] Wang S S, Yang Y H, Fan S S, et al, Chinese Journal of Chromatography, 2020,38(9):1069 34213273 10.3724/SP.J.1123.2020.03001

[63] Wang W F, Zang Y, Yang J L. Chinese Journal of Chromatography, 2020,38(10):1232 34213121 10.3724/SP.J.1123.2020.03029

[64] Shen Y T, Kang J W, Chinese Journal of Chromatography, 2020,38(10):1238 34213122 10.3724/SP.J.1123.2020.05032

[65] Zhang M, Wang Y C, M Atif, et al. Chinese Journal of Chromatography, 2020,38(9):1085 34213275 10.3724/SP.J.1123.2020.02027

[66] Zhao L Y, Lü W J, Niu X Y, et al. Chinese Journal of Chromatography, 2020,38(9):1095 34213276 10.3724/SP.J.1123.2020.02031

[67] Liu L L, Qiao J, Zhang H Y, et al. Chinese Journal of Chromatography, 2020,38(9):1107 34213278 10.3724/SP.J.1123.2020.01006

